# Is Saffron Able to Prevent the Dysregulation of Retinal Cytokines Induced by Ocular Hypertension in Mice?

**DOI:** 10.3390/jcm10214801

**Published:** 2021-10-20

**Authors:** José A. Fernández-Albarral, Miguel A. Martínez-López, Eva M. Marco, Rosa de Hoz, Beatriz Martín-Sánchez, Diego San Felipe, Elena Salobrar-García, Inés López-Cuenca, María D. Pinazo-Durán, Juan J. Salazar, José M. Ramírez, Meritxell López-Gallardo, Ana I. Ramírez

**Affiliations:** 1 Instituto de Investigaciones Oftalmológicas Ramón Castroviejo, Grupo UCM 920105, Universidad Complutense de Madrid, 28040 Madrid, Spain; joseaf08@ucm.es (J.A.F.-A.); rdehoz@med.ucm.es (R.d.H.); elenasalobrar@med.ucm.es (E.S.-G.); inelopez@ucm.es (I.L.-C.); jjsalazar@med.ucm.es (J.J.S.); ramirezs@med.ucm.es (J.M.R.); 2 Departamento de Fisiología, Facultad de Medicina, Grupo UCM 951579, Universidad Complutense de Madrid, 28040 Madrid, Spain; miguma19@ucm.es (M.A.M.-L.); beatrm14@ucm.es (B.M.-S.); diesanfe@ucm.es (D.S.F.); 3 Departamento de Genética, Facultad de CC. Biológicas, Fisiología y Microbiología, Grupo UCM 951579, Universidad Complutense de Madrid, 28040 Madrid, Spain; emmarco@bio.ucm.es; 4 Departamento de Inmunología, Facultad de Óptica y Optometría, Oftalmología y ORL, IdISSC, Universidad Complutense de Madrid, 28040 Madrid, Spain; 5 Ophthalmic Research Unit “Santiago Grisolía”—FISABIO and Cellular and Molecular Ophthalmobiology Unit, University of Valencia, 46017 Valencia, Spain; dolores.pinazo@uv.es; 6 Departamento de Inmunología, Facultad de Medicina, Oftalmología y ORL, IdISSC, Universidad Complutense de Madrid, 28040 Madrid, Spain

**Keywords:** cytokines, BDNF, VEGF, fractalkine, glaucoma, ocular hypertension, microglia, saffron, crocin, retinal glial cells

## Abstract

Cytokine- and chemokine-mediated signalling is involved in the neuroinflammatory process that leads to retinal ganglion cell (RGC) damage in glaucoma. Substances with anti-inflammatory properties could decrease these cytokines and chemokines and thus prevent RGC death. The authors of this study analysed the anti-inflammatory effect of a hydrophilic saffron extract standardized to 3% crocin content, focusing on the regulation of cytokine and chemokine production, in a mouse model of unilateral laser-induced ocular hypertension (OHT). We demonstrated that following saffron treatment, most of the concentration of proinflammatory cytokines (IL-1β, IFN-γ, TNF-α, and IL-17), anti-inflammatory cytokines (IL-4 and IL-10), Brain-derived Neurotrophic Factor (BDNF), Vascular Endothelial Growth Factor (VEGF), and fractalkine were unaffected in response to laser-induced OHT in both the OHT eye and its contralateral eye. Only IL-6 levels were significantly increased in the OHT eye one day after laser induction compared with the control group. These results differed from those observed in animals subjected to unilateral OHT and not treated with saffron, where changes in cytokine levels occurred in both eyes. Therefore, saffron extract regulates the production of proinflammatory cytokines, VEGF, and fractalkine induced by increasing intraocular pressure (IOP), protecting the retina from inflammation. These results indicate that saffron could be beneficial in glaucoma by helping to reduce the inflammatory process.

## 1. Introduction

Glaucoma is a neurodegenerative disease in which there is an irreversible loss of retinal ganglion cells (RGCs), leading to blindness [1]. Intraocular pressure (IOP) is one of the main risk factors for this pathology and the one on which the main current treatments are based [2]. However, IOP control often fails to prevent the progression of the disease, so new factors that could be related to glaucomatous neurodegeneration, such as neuroinflammation, are being discovered [3,4].

In the course of the inflammatory process, retinal glial cells, both microglia and macroglia (Müller cells and astrocytes), can be activated and release factors that can be neuroprotective in some cases and neurodegenerative in others [5,6,7]. Activated microglia can be found along two extreme activation phenotypes, M1 and M2. The M2 phenotype releases neurotrophic factors (neurotrophins, Glial cell line-derived Neurotrophic Factor (GDNF), Brain-derived Neurotrophic Factor (BDNF), etc. and anti-inflammatory cytokines (TGFβ, IL-4, IL-10, etc.) that help control inflammation and promote neuronal survival. However, the M1 phenotype releases proinflammatory mediators (nitric oxide and reactive oxygen species) and proinflammatory cytokines (IL-1β, IL-6, IFN-γ, TNF-α, etc.). Therefore, if this phenotype becomes chronic, it can promote the neurodegenerative process [5,8,9].

In previous studies, we analysed microglial activation [9,10,11,12,13] and the expression of different proinflammatory cytokines (IL-1β, IL-6, IL-17, IFN-γ, TNF-α), anti-inflammatory cytokines (IL-4 and IL-10), BDNF, VEGF, and fractalkine (CX3CL1) [14] in a mouse model of glaucoma at different time points (1, 3, 5, 7, and 15 days) after ocular hypertension (OHT) induction. In this model, we found that the highest microglial activation, as measured by increased cell number, increased number of vertical processes, increased soma size, retraction of the processes, and downregulation of P2RY12 (which is a sensitive marker of the switch from resting to activated microglia [15]), occurred at 3 and 5 days after OHT induction [11]. This peak of microglial activation seemed to overlap with an increased expression of pro-inflammatory cytokines (IL-6 and IFN-γ), anti-inflammatory (IL-4 and IL-10) cytokines, VEGF, fractalkine, and BDNF, which showed greater expression at 1, 3, and 5 days after OHT induction [14]. Higher inflammation was found to occur at these time points, and RGC loss was thereafter triggered; at 3 days after OHT induction, we observed a decrease in the expression of the RGCs marker Brn3a, and from 5 and 7 days onwards, the neurodegenerative process became evident [14,16]. Therefore, 1, 3, and 5 days after OHT induction (especially after 1 and 3 days) would be the ideal time points to analyse whether a substance has anti-inflammatory effects in this murine glaucoma model. Accordingly, we analysed the possible beneficial effect of saffron extracts on the neuroinflammatory and neurodegenerative process in this model of glaucoma, as saffron has important anti-inflammatory, antiapoptotic, and antioxidant properties [17,18,19,20]. The therapeutic activity of saffron may be due to its content in carotenoids, such as crocetin, crocin, and safranal, which are saffron’s main bioactive components, with crocetin being the major component responsible for its therapeutic properties [21,22,23,24]. Different mechanisms of action have been attributed to crocetin including a reduction of proinflammatory molecules and protection against oxidative damage. Crocetin’s anti-inflammatory properties could be due to its capacity to modulate the proinflammatory cytokines’ release from glial cells (IL-6, IL-1β among others); to its ability to block TNF-α expression by microglia, to DNA fragmentation avoidance and thus cell death; to its capacity to modulate the expression of adhesion molecules; to reduce mRNA of some proinflammatory enzymes; and to modulate the NF-κB inflammatory pathway [25,26,27,28,29,30,31]. With regard to its antioxidant properties, crocetin, together with other carotenoids contained in the saffron extract, is able to modulate the expression of genes related to the redox cells system, to inhibit the proteins, and DNA and RNA synthesis; to modify stress markers in the endoplasmic reticulum system; to reduce telomerase activity; and to counteract oxidative stress via tRNA–crocetin union and suppression of the activation of NF-κB through activation of the nuclear factor erythroid 2-related factor 2 (Nrf2) signal related to the modulation of oxidative stress [24,26,32,33,34,35].

We found that at day 3 after OHT induction, animals treated with saffron extracts showed a decrease in the morphological signs of microglial activation and partially reversed the downregulation of P2RY12. Furthermore, they prevented the death of RGCs 7 days after OHT induction, indicating that saffron extracts were able to produce an anti-inflammatory and neuroprotective effect against the damage caused by increased IOP [36]. However, the effects of this saffron extract on the release of different inflammatory mediators with anti-inflammatory or proinflammatory properties to promote the protection of RGCs are currently unknown. Therefore, the purpose of the present work was to analyse whether the administration of saffron extracts in a laser-induced OHT model at 1 and 3 days after OHT induction, in which we found increased cytokine expression in OHT eyes, causes changes in the expression of proinflammatory cytokines (IL-1β, IL-6, IL-17, IFN-γ, and TNF-α), anti-inflammatory cytokines (IL-4 and IL-10), BDNF, VEGF, and fractalkine using multiplex and immunohistochemical techniques.

## 2. Materials and Methods

### 2.1. Animals

The study was performed on male Swiss mice weighing 40–45 g and aged 12–16 weeks, obtained from Charles River Laboratories (Barcelona, Spain). The animals were kept in the animal house of the Faculty of Medicine of the Universidad Complutense de Madrid (Spain). The animals were placed in cages in which the light intensity varied between 9 and 12 lux and the light/dark cycles were 12 h. The animals were fed a standard diet and had free access to it and water. The experimental protocols complied with all of the ethical guidelines endorsed by Spanish law and the Guidelines for Humane Endpoints for Animals Used in Biomedical Research. The study was approved by the Ethics Committee for Animal Research of Complutense University (Madrid, Spain) and the Directorate General of Agriculture and Food, Ministry of Economy and Employment of the Community of Madrid (approval ID number: ES280790000086, 1 April 2016). Finally, the procedures also followed institutional guidelines, the European Union regulations for the use of animals in research, and the Association for Research in Vision and Ophthalmology (ARVO) statement for the use of animals in ophthalmic and vision research.

### 2.2. Experimental Groups

The animals were divided into three study groups: (i) a naïve age-matched control group that received saffron extract (saffron-naïve group; SNG) and (ii) two groups of laser-treated animals that were photocoagulated with laser in the left eye to provoke an OHT and that were treated with saffron extract. These groups were sacrificed 1 (saffron laser-induced group sacrificed on day 1, SLG1d) and 3 days (saffron laser-induced group sacrificed on day 3, SLG3d) after OHT induction. In the laser groups, both saffron OHT eyes (SOHT) and their normotensive saffron-contralateral eyes (S-contralateral) were analysed.

### 2.3. Treatment with Saffron

The company Pharmactive Biotech Products S.L. (Madrid, Spain) was responsible for supplying the hydrophilic stigma extracts of saffron (Crocus sativus L.) that were marketed under the saffron ^®^ EYE brand (Pharmactive Biotech Products S.L., Madrid, Spain) [37,38]. These extracts were standardised to 3.14% total crocin and contain dextrin as a carrier. They were prepared by a proprietary method and analysed by high performance liquid chromatograph (HPLC) (Agilent Technologies 1220, Hewlett-Packard-Strasse, Waldbronn, Germany) [37]. The extract was in powder form and was stored in the dark until use. The dose selected and the time of administration was the same as that used in a previous work [36]. The dose was 60 mg/kg b.w. per day, which contained a total amount of crocin of 1.8 mg. Saffron extract was administered for 15 days in the SNG and 15 days before OHT induction in the laser groups. The administration of saffron extract was maintained after laser treatment until the day of animal sacrifice: 1 day (SL1d) and 3 days (SL3d). At the beginning of the study, in order to calculate the proportion of extract per kilogram of body weight, the animals were weighed. To ensure that the animals received their exact dose, the extract was diluted in water and administered by gavage (0.01 mL/g b.w.).

### 2.4. Anaesthetics

To avoid animal suffering, surgical procedures were performed after the intraperitoneal administration of a general anaesthetic consisting of a mixture of medetomidine (0.26 mg/kg; Medetor^®^, Virbac España S.A., Barcelona, Spain) and ketamine (75 mg/kg; Anesketin^®^, Dechra Veterinary Products SLU, Barcelona, Spain). After the procedure, an ointment containing tobramycin (Tobrex^®^; Alcon, Barcelona, Spain) was administered on to the eyes to prevent eye drying and infection. Subsequently, the animals were returned to their cages for recovery from anaesthesia. An overdose of sodium pentobarbital (Dolethal Vetoquinol^®^, Especialidades Veterinarias, Alcobendas, Madrid, Spain) was used for the sacrifice of the animals. For the measurement of IOP, the animals were given inhalational anaesthesia of 2% isoflurane in oxygen (ISOFLO Isoflurane 100% *w/w*, Zoetis SL, Alcobendas, Madrid, Spain).

### 2.5. Laser Treatment and Measurement of IOP

A laser treatment was performed to induce an OHT. For this purpose, the left eyes of previously anaesthetised animals were treated with a diode laser according to previously described protocols [39,40]. Briefly, the laser beam was applied directly, without any lens, on the episcleral and limbal veins, so that 55–76 burns were performed. The used parameters were: a spot size of 50–100 µm, a duration of 0.5 s, and a power of 0.3 W. IOP was measured in both eyes of all of the experimental groups using a rebound tonometer (Tono-Lab, Tiolat, OY, Helsinki, Finland) [41,42]. IOP was measured after the administration of anaesthesia and always at the same time point (around 9 a.m.) to avoid changes due to circadian rhythms [43]. Six consecutive IOP measurements were performed and averaged each time. In the control group (SNG), IOP was measured before sacrifice. In the SLGs, IOP was measured before laser induction. In the SLG1d group, IOP was also measured on day 1 after laser induction, and in the SLG3d group, it was measured on days 1, 2, and 3 after laser induction.

### 2.6. Multiplexed Immunoassay Study

#### 2.6.1. Protein Assay

For the multiplex immunoassay, animals were sacrificed with an overdose of sodium pentobarbital, after which the retinas were removed and frozen. As in previous studies [14], three retinas were pooled to perform the assay because of the small amount of protein obtained per mouse retina. Thus, four samples per experimental group were assayed, each one coming from three pooled retinas from eyes from the same experimental group. Retinas were homogenized on ice with a lysis buffer (MILLIPLEX MAP Lysis buffer for Multiplexing, Merck KGaA, Darmstadt, Germany) at a ratio of 1: 3 (*w/v*) and then frozen overnight at −70 °C. The following day, the samples were centrifuged (12,000× *g* for 5 min at 4 °C), and the supernatants were collected and processed again in the same way. These last supernatants were aliquoted to analyse the protein concentration by using the Bradford protein assay (Bio-RadDye Reagent Concentrate, Bio-Rad Laboratories, Irvine, CA, USA), which was then measured by a Multiskan reader (Thermo Fisher Technologies, Madrid, Spain). The obtained protein concentration was suitable for immunoassay.

#### 2.6.2. Multiplexed Magnetic Bead Immunoassay

Using two multiplexed magnetic bead immunoassay kits (MILLIPLEX MAP Mouse Cytokine/Chemokine Magnetic Bead Panel; MILLIPLEX MAP Myokine Magnetic Bead Panel, Merck KGaA, Darmstadt, Germany), based on Luminex© technology, we measured the cytokines and myokines chosen for the study in duplicate. Briefly, the followed protocol used magnetic beads (25 µL) that were conjugated with the specific antibodies for the different cytokines/myokines (IFN-γ, IL-1β, IL-4, IL-6, IL-10, IL-17, TNF-α, VEGF, BDNF, and then fractalkine (CX3CL1)) were incubated together with the tissue samples (25 µL) under agitation overnight at 4 °C. After this, the wells were washed three times using a wash buffer. They were then incubated with a biotinylated antibody for 1 h at room temperature. The beads were then incubated for 30 min at room temperature with streptavidin-PE (phycoerythrin), which is a reporter molecule that completes the reaction on the surface of each microsphere. The samples were then washed three times, and the detection component included in the immunoassay kit was added. After immunoassay completion, the samples were analysed using the Bio-Plex suspension array system 200, and the mean fluorescence intensity was measured using Bio-Plex Manager Software 4.1 (Bio-Rad Laboratories, Irvine, CA, USA).

#### 2.6.3. Immunostaining

In order to locate the cells that expressed the factors and cytokines detected in the multiplex assay, we performed an immunohistochemical analysis. As Fernández-Albarral et al. (2021) described [14], each animal was anaesthetised, then transcardially perfused by first employing a 0.9% saline solution and then 4% paraformaldehyde (PFA 4%) in a 0.1 M phosphate buffer. Then, eyes were dissected and fixed for 24 h at 4 °C in PFA. After 24 h, corneas and lenses were removed, and the optical cups containing the retina stood overnight at 4 °C in PFA again. The next day, each eye was washed in phosphate-buffered saline (PBS), pH 7.2, for 30 min. This procedure was repeated 3 times, and then each eye stood in PBS with 11% sucrose at 4 °C for 24 h in order to start the cryoprotection process. Then, the eyes passed to 33% sucrose PBS at 4 °C for 48 h. After the cryoprotection process was finished, the eyes were embedded in a tissue-freezing medium (Tissue-Tek^®^ O.C.T.TM Compound, Sakura Finetek Spain, Barcelona, Spain). During the inclusion, the eye anatomical references were noted to keep track of each part of the retinas. The samples were frozen at −30 °C till they were later used and processed.

For immunostaining techniques, optical cups with retinas were frozen sectioned using a Leica CM-3050 cryostat (Leica Biosystems, Heidelberger, Germany) in 16-μm-thick serial sagittal sections from the nasal to temporal retina. Tissue sections were collected onto gelatine-coated slides (two sections per slide), air-dried, and stored at −30 °C until use. We selected those retinas (OHT and/or contralateral) and time points where the expression of cytokines and factors analysed by multiplex were greatest.

In the present study, we employed primary antibodies (Table 1) against IL-1β, IL-4, IL-6, IL-10, IL-17, INF-Y, fractalkine, TNF-α, and BDNF, which were co-located with Iba-1, a microglial marker; Glial fibrillary Acidic Protein (GFAP), a macroglial marker, and NF-200 (200 kD neurofilament), an RGC axon marker. Each secondary antibody was conjugated with a determined fluorochrome, as indicated in Table 1, which allowed for their detection during a double-labelling fluorescent immunohistochemistry study.

Slides were allowed to dry at room temperature for 60 min in order to increase the adhesion of the slices to the slides. All washes were conducted in PBS, pH 7.2, containing 0.1% Triton X-100, which constituted the washing buffer (WB); incubations were performed in WB with 1% universal serum and the antibody in the desired concentration. After three washes in WB, sections were incubated overnight at 4 °C with the primary antibodies (see Table 1), then rinsed three times in WB, and incubated for 2 h at room temperature with the secondary antibodies, except for Iba-1 (not needed). The details and dilutions of all of the primary and secondary antibodies used are presented in Table 1.

After incubations, sections were washed three more times with WB, then cover-slipped with a Vectashield Vibrance Antifade^®^ mounting medium with DAPI (Ref. H-1800; Vector Laboratories, Burlingame, CA, USA).

Immunostaining batches contained slides of every animal from every experimental group (*n* = 4 per experimental group) as well as an internal control (omitting the primary antibody) to check the specificity of the immunoreaction and to rule out nonspecific binding. Three different batches were run for each primary antibody.

Immunostained slides were observed under a Zeiss Axio Imager M.2 fluorescence microscope (Carl Zeiss AG, Oberkochen, Germany) associated with the Apotome-2 module (Carl Zeiss AG, Oberkochen, Germany) and AxioCam 503 Mono high-resolution camera (Carl Zeiss AG, Oberkochen, Germany). The microscope was equipped with a Zeiss 10 filter set for Alexa Fluor 488, a Zeiss 64 filter set for Alexa Fluor 594, and a 49 filter set for Alexa Fluor 405. Taken images were analysed using ZEN2 software (Carl Zeiss AG, Oberkochen, Germany). All lighting conditions and magnifications were kept constant during the capture process. Figures were prepared using Adobe Photoshop CS4 Extended 10.0 (Adobe Systems, San Jose, CA, USA).

### 2.7. Statistical Analysis

The results obtained both in the IOP measurements and the multiplex analysis were analysed using SPSS version 25 (IBM, Armonk, NY, USA) and were reported as the mean (±standard deviation; SD). If the results followed a normal distribution and equal variances, we performed an ANOVA and then a post-hoc Bonferroni comparison analysis in order to establish significant differences between experimental groups.

If data did not follow a normal distribution and were not homoscedastic, they were transformed (only for IL-6, IL-17, and BDNF analysis). Outliers were deleted according to the SPSS program [56]. Otherwise, non-parametric analyses—in particular, Kruskal–Wallis analysis followed by Mann–Whitney comparisons as a post-hoc—were performed. Only *p*-values under 0.05 were considered statistically significant.

## 3. Results

### 3.1. Intraocular Pressure

**Figure 1:**
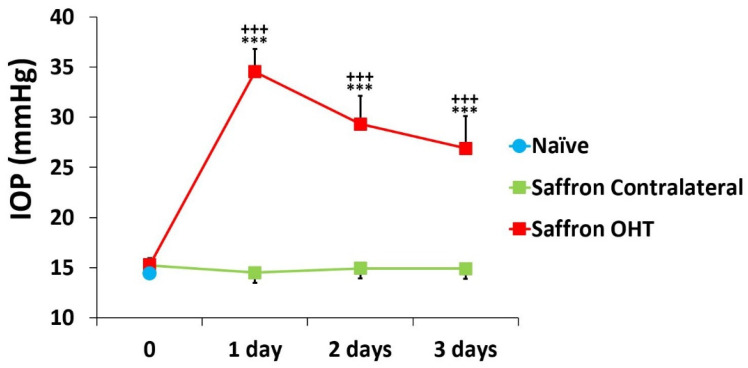
Intraocular pressure (IOP) values after laser-induced ocular hypertension (OHT) in 1, 2, and 3 days after laser OHT induction in saffron-naïve eyes, saffron ocular hypertension eyes (Saffron OHT), and saffron-contralateral eyes (saffron-contralateral). Statistical significance indicators: *** *p* < 0.001 vs. saffron-naïve; +++ *p* < 0.001 vs. saffron-contralateral.

Eyes that had undergone OHT induction (saffron OHT eyes) had significant differences in IOP compared with SNG and saffron-contralateral eyes. (all *p* < 0.001; Figure 1). At all of the analysed time points, the contralateral eyes had an IOP similar to that of the naïve eyes (*p* > 0.05; Figure 1).

### 3.2. Multiplex Analysis

**Figure 2:**
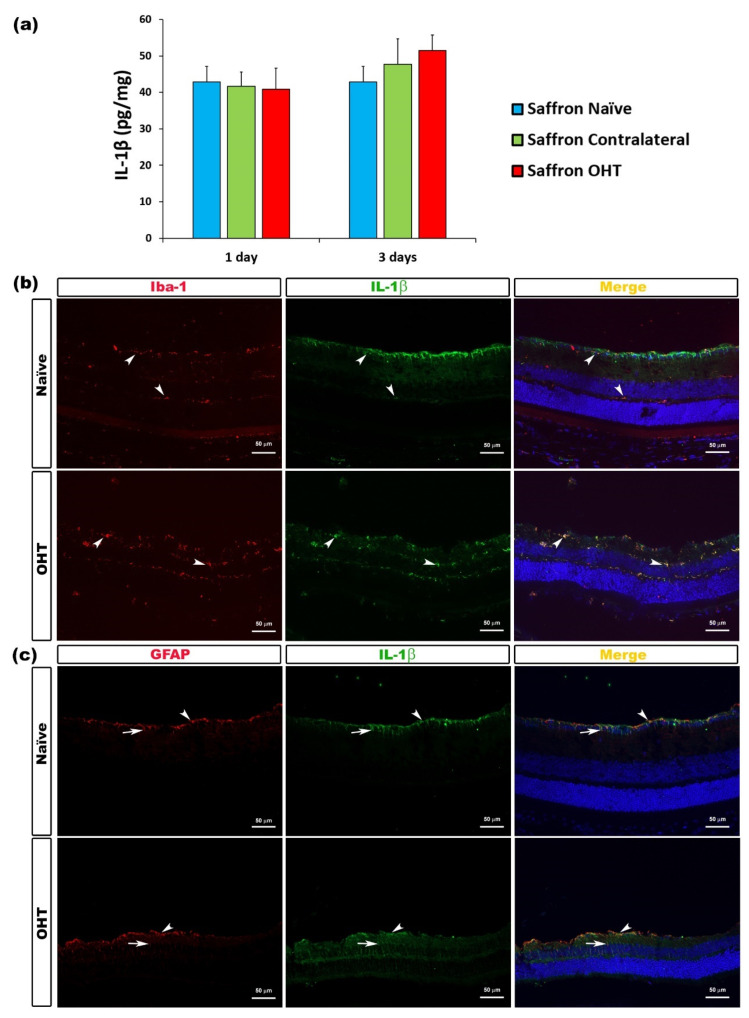
IL-1β levels at 1 and 3 days after laser-induced ocular hypertension (OHT). (**a**) The IL-1β values obtained in the multiplex assay. The histogram shows the mean levels (± SD) of IL-1β (pg/mg) at days 1 and 3 after laser OHT induction, in saffron ocular hypertension eyes (saffron OHT), saffron-contralateral eyes (saffron-contralateral), and naïve eyes. (**b**,**c**) Immunohistochemical study of IL-1β expression in naïve and saffron OHT eyes three days after unilateral laser-induced OHT. Retinal sections were immunolabeled with antibodies to IL-1β (green), Iba-1 (red in (**b**)), or GFAP (red in (**c**)). The merge section is denoted with the colour yellow. (**b**) The arrowhead shows the co-expression of Iba-1 and IL-1β. (**c**) Arrowhead (astrocytes) and arrow (Müller cells) indicate the co-expression of GFAP and IL-1β. Abbreviations: OHT (ocular hypertension); IL-1β (interleukin 1 beta); Iba-1 (ionized calcium-binding adaptor molecule); GFAP (glial fibrillary acidic protein).

Among the measured proinflammatory cytokines, 1 and 3 days after OHT induction, no significant differences were found in IL-1β expression between saffron OHT and saffron contralateral eyes compared to saffron-naïve eyes. Thus, retinal IL-1β levels were not affected by the laser OHT induction (Figure 2).

**Figure 3:**
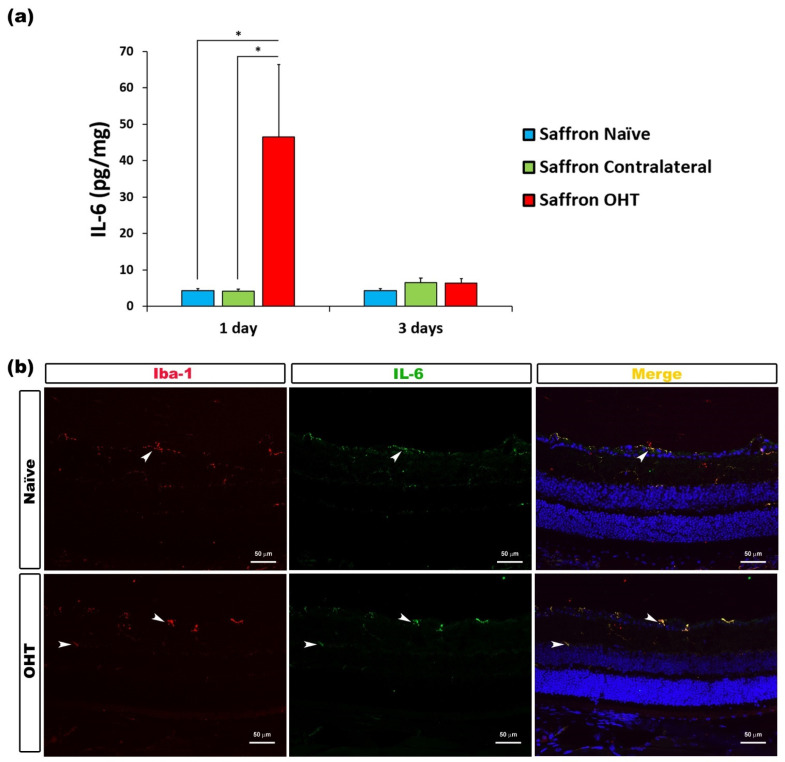
IL-6 levels at 1 and 3 days after laser-induced ocular hypertension (OHT). (**a**) The IL-6 values obtained in the multiplex assay. The histogram shows the mean levels (±SD) of IL-6 (pg/mg) at days 1 and 3 after laser OHT induction in saffron ocular hypertension eyes (saffron OHT), saffron-contralateral eyes (saffron-contralateral), and saffron-naïve eyes. Statistical significance indicators: * *p* < 0.05. (**b**) Immunohistochemical study of IL-6 expression in saffron OHT eyes one day after unilateral laser-induced OHT. Retinal sections were immunolabeled with antibodies to IL-6 (green) and Iba-1 (red). Merge is denoted by the colour yellow. The arrowhead shows the co-expression of Iba-1 and IL-6 in saffron OHT and saffron-naïve eyes. Abbreviations: OHT (ocular hypertension); IL-6 (interleukin 6); Iba-1 (ionized calcium-binding adaptor molecule).

Regarding the IL-6 content, 1 day after OHT laser induction, a significant increase in its expression was observed in saffron OHT eyes compared to saffron contralateral eyes and saffron-naïve eyes (all *p* < 0.05). No significant differences were observed for IL-6 expression 3 days after OHT laser induction. Thus, retinal IL-6 expression was significantly higher exclusively 1 day after the laser induction in the saffron OHT eyes; cytokine levels were then returned to normal values 3 days post-laser treatment (Figure 3).

**Figure 4:**
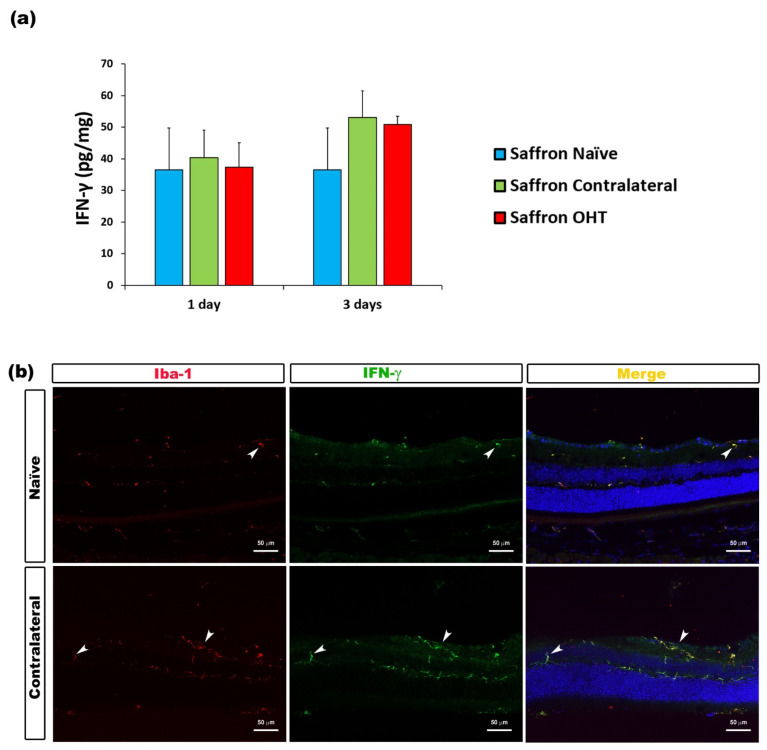
IFN-γ levels at 1 and 3 days after laser-induced ocular hypertension (OHT). (**a**) The IFN-γ values obtained in the multiplex assay. The histogram shows the mean levels (±SD) of IFN-γ (pg/mg) at days 1 and 3 after laser OHT induction in saffron ocular hypertension eyes (saffron OHT), saffron-contralateral eyes (saffron-contralateral), and saffron-naïve eyes. (**b**) Immunohistochemical study of IFN-γ expression in saffron-contralateral eyes three days after unilateral laser-induced OHT. Retinal sections were immunolabeled with antibodies to IFN-γ (green) and Iba-1 (red). Merge is denoted with the colour yellow. The arrowhead shows the co-expression of Iba-1 and IFN-γ in saffron-contralateral and saffron-naïve eyes. Abbreviations: OHT (ocular hypertension); IFN-γ (interferon-γ); Iba-1 (ionized calcium-binding adaptor molecule).

**Figure 5:**
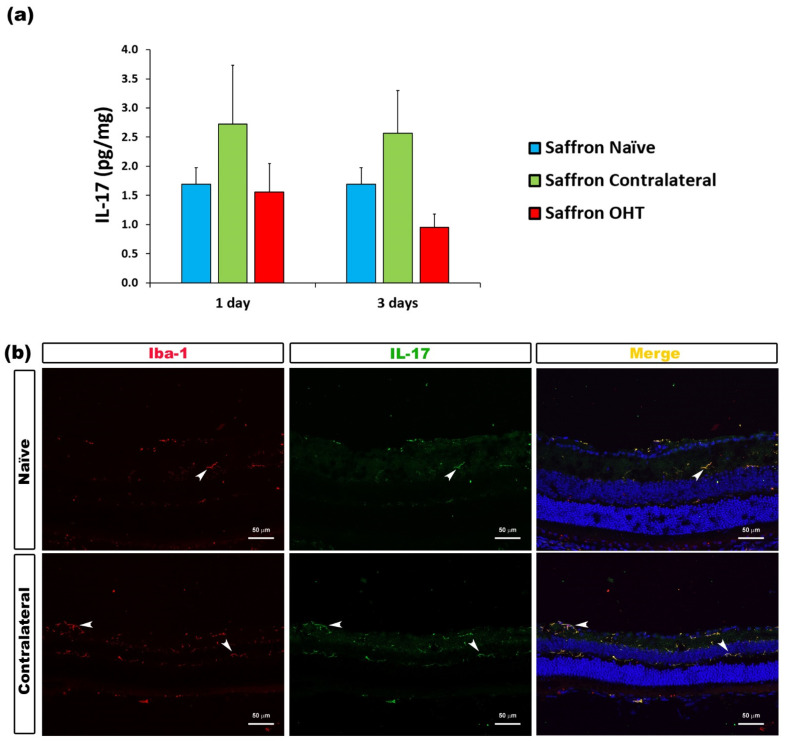
IL-17 levels at 1 and 3 days after laser-induced ocular hypertension (OHT). (**a**) The IL-17 values obtained in the multiplex assay. The histogram shows the mean levels (±SD) of IL-17 (pg/mg) at days 1 and 3 after laser OHT induction in saffron ocular hypertension eyes (saffron OHT), saffron-contralateral eyes (saffron-contralateral), and naïve eyes. (**b**) Immunohistochemical study of IL-17 expression in saffron-contralateral eyes one day after unilateral laser-induced OHT. Retinal sections were immunolabeled with antibodies to IL-17 (green) and Iba-1 (red). Merge is denoted by the colour yellow. The arrowhead shows the co-expression of Iba-1 and IL-17 in saffron-contralateral and saffron-naïve eyes. Abbreviations: OHT (ocular hypertension); IL-17 (interleukin-17); Iba-1 (ionized calcium-binding adaptor molecule).

The analysis of IFN-γ levels showed no significant differences after experimental OHT induction. IFN-γ levels were not affected by the laser induction at any of the time-points considered (Figure 4). Similarly, the analysis of IL-17 levels showed no significant differences after laser induction in comparison to saffron-naïve eyes. Retinal IL-17 levels were not affected by the experimental OHT induction in the saffron OHT eyes and neither in saffron-contralateral eyes (Figure 5).

**Figure 6:**
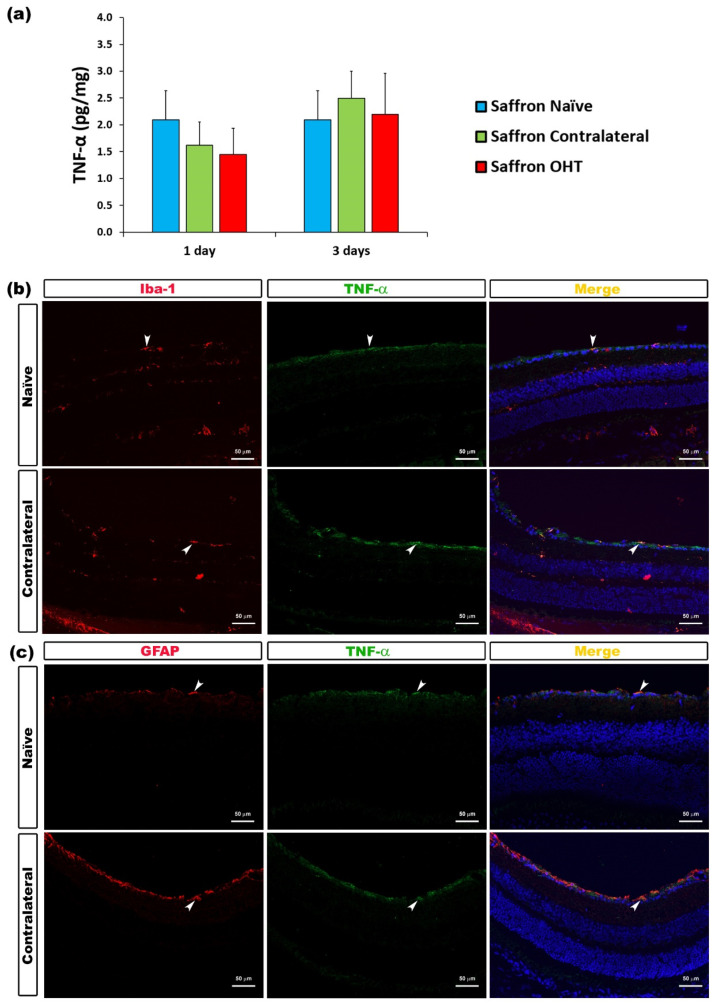
TNF-α levels at 1 and 3 days after laser-induced ocular hypertension (OHT). (**a**) The TNF-α values obtained in the multiplex assay. The histogram shows the mean levels (±SD) of TNF-α (pg/mg) at days 1 and 3 after laser OHT induction in saffron ocular hypertension eyes (saffron OHT) and saffron-contralateral eyes (saffron-contralateral) as well as in saffron-naïve eyes. (**b**) Immunohistochemical study of TNF-α expression in saffron-contralateral eyes three days after unilateral laser-induced OHT. Retinal sections were immunolabeled with antibodies to TNF-α (green) and Iba-1 (red in (**b**)), or GFAP (red in (**c**)). Merge is denoted by the colour yellow. (**b**) The arrowhead shows the co-expression of Iba-1 and TNF-α. (**c**) The arrowhead shows the co-expression of GFAP and TNF-α. Abbreviations: OHT (ocular hypertension); TNF-α (tumour necrosis factor-α); Iba-1 (ionized calcium-binding adaptor molecule); GFAP (glial fibrillary acidic protein).

Regarding TNF-α levels, 1 and 3 days after OHT induction, no significant changes were found in its expression neither in saffron OHT eyes nor in saffron contralateral eyes when compared to saffron-naïve eyes. Retinal TNF-α levels were not affected by the experimental OHT induction (Figure 6).

**Figure 7:**
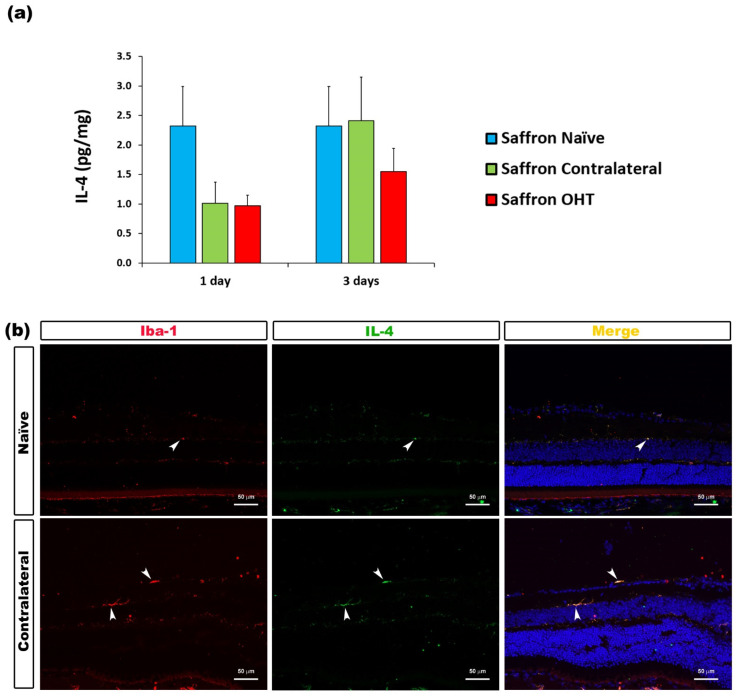
IL-4 levels at 1 and 3 days after laser-induced ocular hypertension (OHT). (**a**) The IL-4 obtained in the multiplex assay. The histogram shows the mean levels (±SD) of IL-4 (pg/mg) at days 1 and 3 after laser OHT induction in saffron ocular hypertension eyes (saffron OHT) and saffron-contralateral eyes (saffron-contralateral) as well as in naïve eyes. (**b**) Immunohistochemical study of IL-4 expression in saffron-contralateral eyes three days after unilateral laser-induced OHT. Retinal sections were immunolabeled with antibodies to IL-4 (green) and Iba-1 (red). Merge is denoted by the colour yellow. The arrowhead shows the co-expression of Iba-1 and IL-4 in saffron-contralateral and saffron-naïve eyes. Abbreviations: OHT (ocular hypertension); IL-4 (interleukin- 4); Iba-1 (ionized calcium-binding adaptor molecule).

The analysis of the anti-inflammatory cytokines showed that 1 and 3 days after OHT induction, IL-4 expression was not significantly affected by the laser treatment in saffron-treated eyes, neither in OHT nor in their contralateral eyes, compared to saffron-naïve eyes (Figure 7).

**Figure 8:**
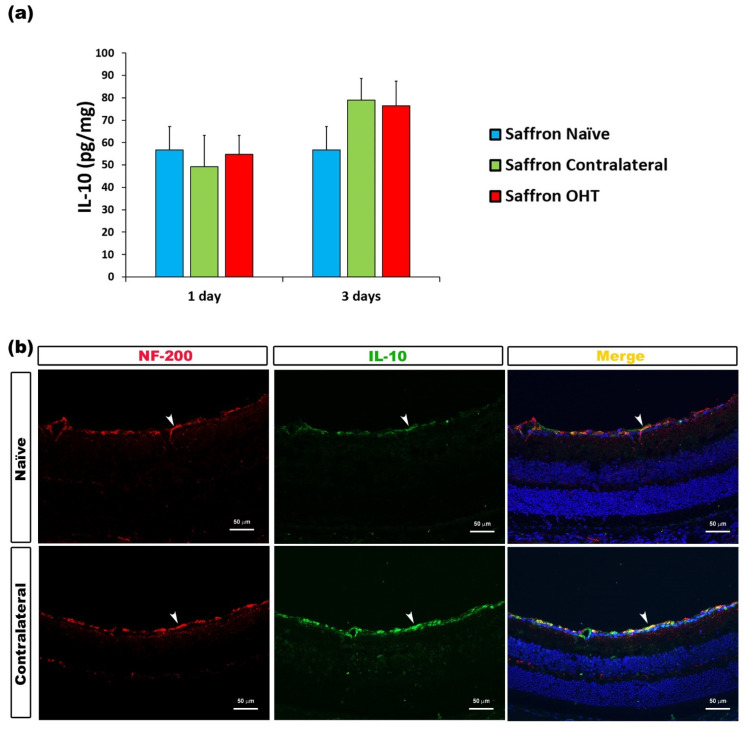
IL-10 levels at 1 and 3 days after laser-induced ocular hypertension (OHT). (**a**) The IL-10 values obtained in the multiplex assay. The histogram shows the mean levels (±SD) of IL-10 (pg/mg) at days 1 and 3 after laser OHT induction in saffron ocular hypertension eyes (saffron OHT) and saffron-contralateral eyes (saffron-contralateral) as well as in naïve eyes. (**b**) Immunohistochemical study of IL-10 expression in saffron-contralateral eyes three days after unilateral laser-induced OHT. Retinal sections were immunolabeled with antibodies to IL-10 (green) and NF-200 (red). Merge is denoted by the colour yellow. The arrowhead shows the co-expression of Iba-1 and IL-10 in saffron-contralateral and saffron-naïve eyes. Abbreviations: OHT (ocular hypertension); IL-10 (interleukin-10); NF-200 (neurofilament-200 KDa).

Regarding IL-10, at all time-points studied after OHT induction, no significant differences were found. IL-10 expression was not altered in saffron OHT and saffron contralateral eyes compared to saffron-naïve eyes; retinal IL-10 levels were not affected by the laser treatment in saffron-treated animals (Figure 8).

**Figure 9:**
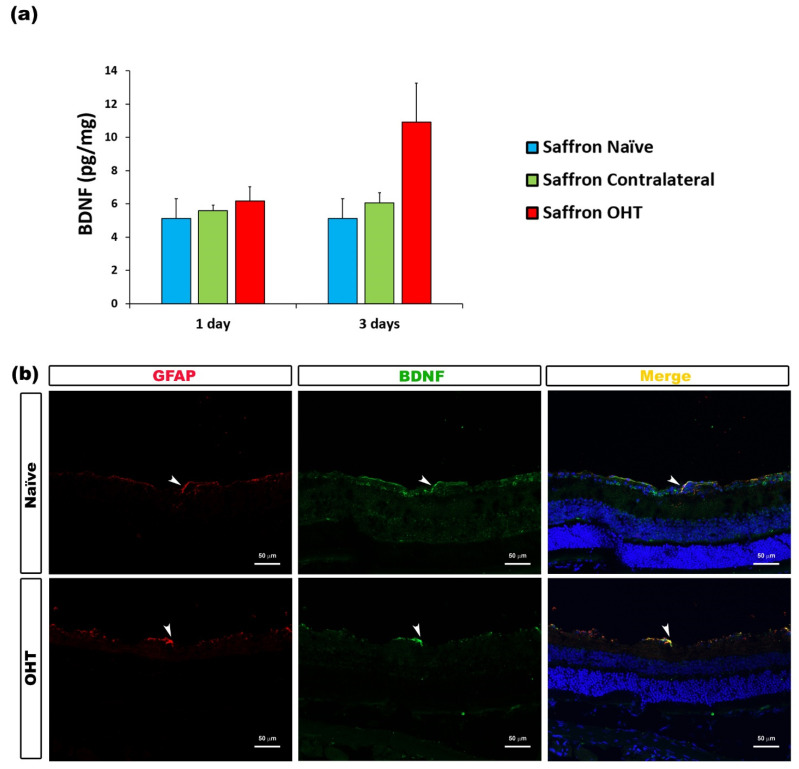
BDNF levels at 1 and 3 days after laser-induced ocular hypertension (OHT). (**a**) The BDNF values obtained in the multiplex assay. The histogram shows the mean levels (± SD) of BDNF (pg/mg) at days 1 and 3 after laser OHT induction in saffron ocular hypertension eyes (saffron OHT), saffron-contralateral eyes (saffron-contralateral), and naïve eyes. (**b**) Immunohistochemical study of BDNF expression in saffron OHT eyes three days after unilateral laser-induced OHT. Retinal sections were immunolabeled with antibodies to BDNF (green) and GFAP (red). Merge is denoted by the colour yellow. The arrowhead shows the co-expression of GFAP and BDNF in saffron OHT and saffron-naïve eyes. Abbreviations: OHT (ocular hypertension); BDNF (brain-derived neurotrophic factor); GFAP (glial fibrillary acidic protein).

Regarding the analysed neurotrophic factors, BDNF levels showed no significant changes among the saffron groups after experimental OHT induction. Retinal BDNF levels were not affected by the laser treatment at 1 or 3 days post OHT laser-induction (Figure 9).

**Figure 10:**
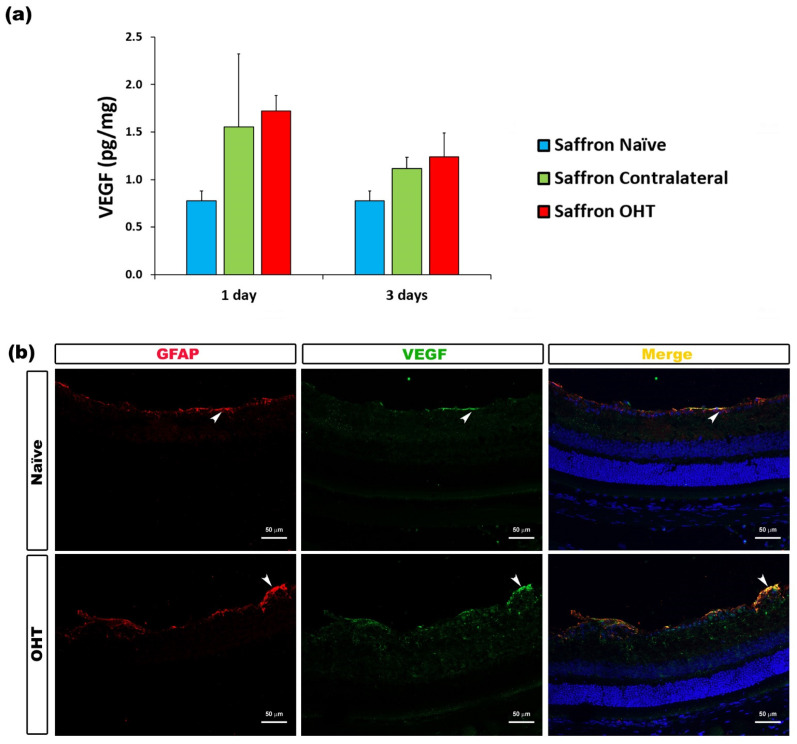
VEGF levels at 1 and 3 days after laser-induced ocular hypertension (OHT). (**a**) The VEGF values obtained in the multiplex assay. The histogram shows the mean levels (±SD) of VEGF (pg/mg) at days 1 and 3 after laser OHT induction in saffron ocular hypertension eyes (saffron OHT) and saffron-contralateral eyes (saffron-contralateral) as well as in saffron-naïve eyes. (**b**) Immunohistochemical study of VEGF expression in saffron OHT eyes one day after unilateral laser-induced OHT. Retinal sections were immunolabeled with antibodies to VEGF (green) and GFAP (red). Merge is denoted by the colour yellow. The arrowhead shows the co-expression of GFAP and VEGF in saffron OHT and saffron-naïve eyes. Abbreviations: OHT (ocular hypertension); VEGF (vascular endothelial growth factor); GFAP (glial fibrillary acidic protein).

The analysis of VEGF expression rendered no significant differences between saffron-naïve eyes and saffron OHT and saffron-contralateral eyes after laser treatment. Retinal VEGF levels were not affected by the experimental OHT induction in these saffron-treated animals (Figure 10).

**Figure 11:**
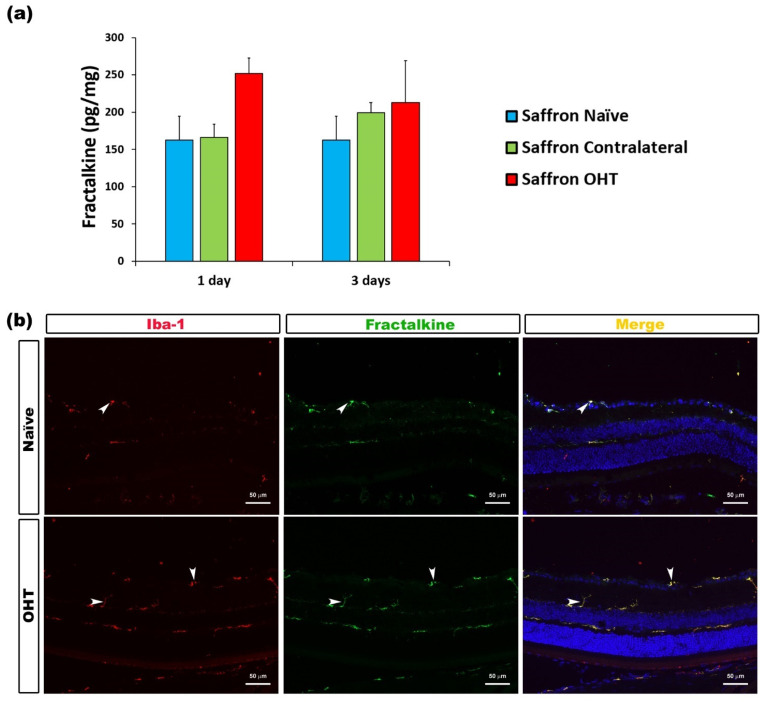
Fractalkine levels at 1 and 3 days after laser-induced ocular hypertension (OHT). (**a**) The fractalkine values obtained in the multiplex assay. The histogram shows the mean levels (±SD) of fractalkine (pg/mg) at days 1 and 3 after laser OHT induction in saffron ocular hypertension eyes (saffron OHT) and saffron-contralateral eyes (saffron-contralateral) as well as in saffron-naïve eyes. (**b**) Immunohistochemical study of fractalkine expression in saffron OHT eyes one day after unilateral laser-induced OHT. Retinal sections were immunolabeled with antibodies to fractalkine (green) and Iba-1 (red). Merge is denoted by the colour yellow. The arrowhead shows the co-expression of Iba-1 and fractalkine in saffron OHT and saffron-naïve eyes. Abbreviations: OHT (ocular hypertension); Iba-1 (ionized calcium-binding adaptor molecule).

The analysis of the content of the microglial activator factor fractalkine (Figure 11) 1 and 3 days after OHT induction revealed no significant differences between experimental groups. Retinal fractalkine levels were not affected by the laser treatment at any day post-laser among the saffron-treated animals.

### 3.3. Cytokine Colocalizations with Different Cell Populations

The results obtained in the immunohistochemical assay demonstrated that all of the pro- and anti-inflammatory cytokines as well as BDNF, VEGF, and fractalkine, colocalized with specific retinal cell populations (see Table 2).

## 4. Discussion

In the present study, we demonstrated that following saffron treatment, most of the previously observed changes in the concentration of proinflammatory (IL-1β, IFN-γ, IL-6, and IL-17) cytokines, anti-inflammatory cytokines (IL-4 and IL-10), BDNF, VEGF, and fractalkine in response to laser-induced OHT [14] were absent. Among all of the analysed pro- and anti-inflammatory cytokines, only IL-6 levels were significantly increased in the OHT eye one day after the surgery compared with the naïve control group, although to a much lesser extent. These augmented IL-6 levels returned to normal at day 3 after laser treatment, an effect that might have been related to the changes in IOP. Moreover, the immunofluorescent study allowed us to confirm that the cytokines were located on the same cellular subtypes within the retina, which was in agreement to a previous study that characterized the temporal profile of neuroinflammation in the same experimental model of glaucoma [14]. Specifically, IL-1β, IL-6, IFN-γ, IL-17, TNF-α, IL-4, and fractalkine colocalize with microglia; IL-1β, TNF-α, VEGF, and BDNF colocalize with macroglia; and IL-10 colocalizes with axons of RGCs. Since the saffron extract used in this study is commercialized, as saffron ^®^ EYE with 3.14% of crocin, results could be replicated in future experiments, and its underlying mechanisms further investigated, as well as its potential therapeutic application.

In a previous study, using this experimental model of glaucoma, we were able to prevent RGC death and the associated neuroinflammation (focused on morphological signs of microglial activation and on the expression of P2RY12) following the oral administration of saffron at the same dosage as that used in the current study [36]. Recently, we better characterized the temporal profile of the neuroinflammatory damage, focusing on both the morphological signs of microglial activation [11] and the cytokine expression profile [14] induced in both the OHT and contralateral eye in this animal model of glaucoma. Therefore, in the present research, which was designed as an extension of these three previous studies, we aimed to further investigate the anti-inflammatory properties of saffron in glaucoma while focusing on the cytokine expression profile. This research was performed 1 and 3 days after laser-induced OHT since previous studies had detected microglial activation one day after surgery, and the peak of microglial activation was described on day 3 after lasering [11]. In addition, these two days after OHT induction were also selected as in our previous study because these were the days on which the greatest variation in cytokine expression occurred in both OHT and contralateral eyes [14].

In the previous study [14], we demonstrated that this model of laser-induced OHT led to a loss of cytokine homeostasis in the retina. Similar changes in cytokine levels have also been observed in the heritable DBA/2J model of glaucoma in response to activation of retinal glial cells [57]. Moreover, another natural compound, a Chinese herb extract containing Triptolide, has been shown to exert anti-inflammatory effects in DBA/2J mice, mostly by reducing microglial activation and proinflammatory factors, such as TNF-α, probably due to modulation of NF-κB [58]. Thus, herbal extracts with anti-inflammatory properties, such as saffron, may be effective in the management of glaucomatous eyes not only in our laser-induced OHT murine model, but also in other genetic and/or interventional models. Future studies will evaluate the efficacy of this saffron extract in additional and complimentary animal models.

It has been found that saffron suppresses the secretion of proinflammatory cytokines, such as TNF-α, IL-1β, IL-6, and IFNγ, among others, decreasing inflammation in different organs or systems (such as the nervous system [59,60,61,62,63], respiratory system [64,65], gastrointestinal system [66,67], cardiovascular system [68,69], and urogenital system [70,71]), as observed in various experimental models and in vitro experiments. In the present study, the oral administration of saffron seems to prevent and/or revert the release of these cytokines, possibly as a result of the previously reported decrease in microglial activation [36].

In the current study, no significant differences were observed in IL-1β levels between all groups treated with saffron. However, in the previous study, performed on untreated saffron mice [14], a significant decrease in IL-1β levels was detected in the OHT at 1 day, but an increase in its content in both contralateral (1 day) and contralateral (3 days) eyes was also detected. Discrepant results have been previously reported when using a similar model of laser-induced OHT in which only a modest IL-1β mRNA upregulation, not IL-1β immunolabeling, was observed in the retina. Three hypotheses were then proposed to explain such discrepancies: (i) the absence of mRNA translation and an overly fast protein degradation; (ii) an expression restricted to isolated cells; or (iii) an insufficient amount of protein that did not allow for its detection through the employed assay [72]. The reported downregulation of IL-1β in OHT at 1 day was explained by the actions of IL-4 and IL-10 [14]. IL-4 and IL-10 polarize the M2 microglial phenotype and downregulate the production of IL-1β, following a negative feedback model in order to control the inflammatory response [73]. The results of Chidlow et al. (2012) [72] and Fernández-Albarral et al. (2021) [36] are contradictory to those obtained in this work, but our model demonstrated that either the changes produced by OHT were reversed by saffron treatment or that OHT did not affect the retinal content of IL-1β in the saffron-treated groups. Following the approach of Chidlow et al. (2012) [72], IL-1β expression may have not been high enough to be detected. However, after comparing our results with those obtained by Fernández-Albarral et al. (2021) [14] and considering the possible relationship between the anti-inflammatory cytokines IL-4 with IL-1β, we can state that since our treatment lowered IL-1β levels, a compensatory response of IL-4 might not be further required, thus explaining the absence of significant differences detected in these anti-inflammatory cytokines compared with the control group. Our current results may reinforce this association since in the saffron-treated animals, OHT did not affect the IL-1β retinal content and IL-4 levels did not change.

TNF-α levels did not differ in any group with respect to the control one, and a comparison could not be established with the previous non-saffron model, since no detectable concentration was found in the previously performed multiplex immunoassay. However, TNF-α regulation seemed to follow the same route as the one previously described for IL-1β, which is evidence indicating the preventive role of saffron in neuroinflammation and microglial activation, thus avoiding the release of proinflammatory cytokines and the need for a compensatory anti-inflammatory cytokine release [36,72,73]. Another possible approach could be the one previously stated by Chidlow et al. (2012) [72]: the absence of mRNA translation and an overly fast protein degradation; an expression by isolated cells; or an insufficient amount of protein that did not allow for its detection through the employed assay.

In the present study, as previously indicated, only IL-6 levels were significantly increased in the OHT eye at 1 day. This result further corroborates the increase in IL-6 levels observed in the prior study following OHT on saffron-free animals, despite the reported increase in that study being around 20 times higher (716.28 pg/mg) than among saffron-treated animals (46.53 pg/mg). Thus, saffron once again seems to counteract the effects induced by OHT. This cytokine is one of the main causes of RGC death in glaucoma models, leading to massive optic nerve degeneration that provokes a loss in visual acuity, as Echevarría et al. (2017) [74] demonstrated in a microbead glaucoma mouse model. However, IL-6 has also been proposed to have a neuroprotective effect; in particular, in an in vitro model of increased IOP, IL-6 was reported to counteract the proapoptotic effects of other factors [75]. The relationship between IOP elevation and IL-6 secretions was confirmed in both Fernández-Albarral et al. (2021) [14] and the current study, in which the highest expression of IL-6 occurred 1 day after OHT induction. However, in this study, we observed that despite the fact that pressure values remained elevated 3 days after laser induction, saffron was able to regulate IL-6 values.

IFN-γ showed no significant differences in the present study, but in the study of Fernández-Albarral et al. (2021) [14], it significantly increased in OHT eyes at 3 day. IFN-γ is directly related to microglial activation, polarizing it into the M1 phenotype and inducing the production of IL-1β, IL-6, TNF-α, reactive oxygen species (ROS), and nitric oxide (NO) [76], all of them being part of the inflammatory response. The capacity of saffron to prevent an increase in IFN-γ levels may underlie its ability to reduce microglial activation [14] and, thus (indirectly), its capacity to diminish the production and release of proinflammatory mediators. Its potential action on IFN-γ may be key for the anti-inflammatory properties of saffron.

IL-17 can induce neuroinflammation by recruiting T-helper-17 lymphocytes, which are highly related to the inflammatory response in autoimmune pathologies [77]. As expected, in our current study, non-significant changes in IL-17 levels were detected in both experimental groups, although in our previous study in untreated saffron animals, a downregulation of IL-17 content was detected in OHT eyes 1 and 3 days after surgery. We previously hypothesised this IL-17 decrease is caused by the counteracting effect of some anti-inflammatory cytokines, such as IL-4 and IL-10 [36]. In the present study, no elevations of IL-4 or IL-10 levels were detected, and no changes in IL-17 levels were expected.

In our previous study, we showed an upregulation of BDNF in OHT eyes 1 and 3 days after laser treatment [14], probably due to the fact that BDNF is mainly released after a significant RGC loss. BDNF participates in RGC survival by inhibiting apoptosis, as demonstrated in a rat OHT model [78]. In the current study, BDNF levels were not changed. As microglial activation may have been downregulated thanks to the saffron treatment, the glaucoma-related massive RGC death might not have occurred, thus making BDNF release unnecessary.

In our previous study, in untreated saffron animals, VEGF levels were significantly increased in OHT at 1 day (54.35 pg/mg); however, the VEGF values obtained in the present work in the saffron-treated animals were much lower (1.72 pg/mg). The increased expression of VEGF is associated with an augmentation of vascular permeability and the attraction of phagocytic cells [79]. VEGF directly participates in vasculogenesis, increasing its levels around damaged tissue. The excessive IOP may have exerted damaging effects, or even a disruption of the blood–retinal barrier (BRB), caused by a direct increase in the surrounding VEGF levels [80]. In the current study, no significant differences were observed in VEGF levels in any experimental group, suggesting that saffron may have protected BRB integrity, avoiding damage and/or disruption and thus avoiding the infiltration of hazardous substances and/or specific, potentially harmful cell populations to the retina. Future studies focused on the integrity of the BRB would be of great interest to elucidate whether saffron is able to prevent damage (if the BRB is intact) or counteract the already induced neuroinflammatory response (if the BRB is damaged).

No significant differences were found regarding fractalkine levels. As previously stated, fractalkine is a chemokine secreted by neurons that directly induce the proliferation, activation, and migration of microglia [81,82]. Microglia seems to co-locate with fractalkine because of the fractalkine–receptor (CXCR1) interaction since CXCR1 is located on microglial cells, so fractalkine may remain on the microglial surface. Sokolowski et al. (2014) [83] proposed that apoptotic neurons release fractalkine as a “find me” signal, stimulating the migration of microglial processes towards the apoptotic neurons in order to eliminate debris. In our previous analysis, fractalkine levels were higher in OHT at 1 day, possibly as an initial response to neuronal damage that enhanced early microglial activation and migration [14]. Upon fractalkine increase and consequent microglial activation on OHT at 1 day, microglial cells may remain active through additional signals from the cellular environment (mainly astroglia and Müllers cells) and not just by fractalkine. This may explain the decrease in fractalkine levels in OHT eye at 3 days and contralateral eye at 1 and 3 days, which may not imply a lack of microglial activation. In the current work, saffron prevented the appearance of fractalkine changes, thus suggesting a neuroprotective role for saffron that may initially prevent neuronal apoptosis and subsequent posterior fractalkine release. The maintenance of fractalkine homeostasis gives support to our main hypothesis of a reduction of the inflammatory response, since under normal fractalkine levels, microglial cells might not become active, cytokine release may not occur, and RGC survival might therefore be enhanced.

It is worth mentioning that saffron-dried stigmas have been used in traditional medicine for a long time, but Zeinali et al. (2019) [31] questioned how the saffron affects the immune response, explaining its antioxidant, antiapoptotic, and anti-inflammatory properties with its radical scavenging properties. Saffron acts as a transcription inhibitor for TNF-α, IFN-Y, IL-1β, IL-6, and IL-17, and it downregulates inflammatory enzymes, such as myeloperoxidase, cyclooxygenase-2 (COX-2), inducible nitric oxide synthase (INOS), phospholipase A2, and prostanoids. These authors reviewed different models of inflammation and proposed that saffron may reduce the production of inflammatory mediators by blocking Toll-like receptors (such as TLR2) or their subsequent activation cascade, which leads to the production of inflammatory cytokines and chemokines, enzymes, and growth factors. Saffron blocks the NF-kB pathway, which produces IL-1, IL-2, IFN-Y, COX-2, iNOS, and TNF-α, inflammatory mediators related to carcinogenesis [84,85]. In particular, crocin has been reported to reduce the mRNA expression of TNF-α, IFN-Y, IL-1β, and IL-6, as well as iNOS and COX-2, in the mucosa [85]. Crocin can also inhibit the MAPK pathway, as it was found to block the synthesis of TNF-α, IL-1β, IL-6, and iNOS in the intervertebral discs in an LPS rat model [86]. Moreover, different neuroinflammation models focused on the study of microglia have also demonstrated the anti-inflammatory effects of saffron. Pretreatment with crocin is able to reduce the production of NO, TNF-α, and IL-1β by repressing NF-κB activity [62]. Crocetin was found to decrease the expression of inflammatory cytokines (IL-1β, IL-6, IL-10, TNF-α, iNOS, and COX-2) in BV2 microglial cells [87]. Crocin prevents NF-κB activation and decreases NO, TNF-α, IL-1β, and ROS expression in brain microglial cells after LPS stimulation [27]. All of these previously stated studies reinforce our main hypothesis, since the blocking of the MAPK and NF-kB routes can regulate the production of the above-mentioned inflammatory mediators in different tissues and animal models that can be compared with our OHT-induced glaucoma model. It is important to consider that our study may have certain limitations, such as the limited number of Inflammatory markers evaluated, which would require new studies with a broader and more unbiased proteomic approach in which the mechanistic aspects associated with saffron extracts could be defined.

## 5. Conclusions

In conclusion, this study demonstrates that saffron extract standardized to 3.14% crocin content is effective in regulating the production of proinflammatory cytokines, VEGF, and fractalkine induced by increased IOP, thus protecting the retina from its related damage. In addition, it highlights the relevance of anti-inflammatory treatment to control the inflammatory process generated after ocular hypertension. Saffron extracts could be beneficial as coadjuvant therapies in the treatment of glaucoma, thus helping to decrease the inflammatory process that occurs in this pathology.

## Figures and Tables

**Table 1:** Antibodies employed for the immunostaining analysis and their corresponding concentrations.

Colour	Primary Antibody	Conc.	Secondary Antibody	Conc.
GREEN	Rabbit polyclonal anti-IL-1β (ref. ab9722, Abcam plc) [44]	1:250	Goat anti-rabbit Alexa Fluor 488 (ref. ab150077, Abcam plc)	1:150
	Rabbit polyclonal anti-IL-6 (ref. ab208113, Abcam plc) [45]	1:200		
	Rabbit polyclonal anti-IL-17 (ref. ab79056, Abcam plc) [46]	1:300		
	Rabbit polyclonal anti-IFNγ (ref. ab9657, Abcam plc) [47]	1:300		
	Rabbit monoclonal anti-BDNF (ref. ab213323, Abcam plc) [44]	1:250		
	Rabbit monoclonal anti-VEGF receptor 1 (ref. ab32152, Abcam plc) [48]	1:200		
	Rabbit polyclonal anti-CX3CL1 (ref. ab25088, Abcam plc) [49]	1:500		
	Rabbit polyclonal anti-TNFα (ref. ab9739, Abcam plc) [50]	1:300		
	Rat monoclonal anti-IL-4 (ref. ab11524, Abcam plc) [51]	1:250	Goat anti-rat Alexa Fluor 488 (ref. ab150165, Abcam plc)	1:150
	Rat monoclonal anti-IL-10 (ref. ab189392, Abcam plc) [52]	1:200		
RED	Rabbit polyclonal anti-Iba1 Red Fluorochrome 635 conjugated (ref. 5100756, Wako Chemicals GmbH) [53]	1:200		
	Chicken polyclonal anti-GFAP (ref. AB5541, Sigma-Aldrich) [54]	1:200	Goat anti-chicken IgY (H+L) Alexa Fluor 594 (ref. A-11042, Invitrogen)	1:300
	Rabbit polyclonal anti-NF-200 (ref. N4142, Sigma-Aldrich) [55]	1:150	Donkey anti-rabbit IgG1 Alexa Fluor 594 (ref. A21207, Invitrogen)	1:800

Antibodies used in the study: interleukin 1 beta (IL-1β), interleukin 6 (IL-6), interleukin 17 (IL-17), interferon gamma (IFN-γ), brain-derived neurotrophic factor (BDNF), vascular endothelial growth factor (VEGF), fractalkine (CX3CL1), tumour necrosis factor alpha (TNF-α), interleukin 4 (IL-4), and interleukin 10 (IL-10); also included are the antibodies used to identify microglial cells (Iba-1), axons (NF-200 KDa), and macroglia (GFAP). The colour (green/red) indicates how the immunostaining is labelled.

**Table 2:** Co-location of cytokines with specific cell types.

Cytokine	Co-Expression Cell Type	Figures
IL-1β	Microglia and Macroglia (astrocytes and Müller cells)	Figure 2
IL-6	Microglia	Figure 3
INF-γ	Microglia	Figure 4
IL-17	Microglia	Figure 5
TNF-α	Microglia and Astrocytes	Figure 6
IL-4	Microglia	Figure 7
IL-10	Axons of retinal ganglion cells	Figure 8
BDNF	Macroglia (astrocytes and Müller cells)	Figure 9
VEGF	Macroglia (astrocytes and Müller cells)	Figure 10
Fractalkine	Microglia	Figure 11

The table represents the cells showing the expression of the different cytokines and factors used in this study: interleukin 1 beta (IL-1β), interleukin 6 (IL-6), interleukin 17 (IL-17), interferon gamma (IFN-γ), brain-derived neurotrophic factor (BDNF), vascular endothelial growth factor (VEGF), fractalkine (CX3CL1), tumour necrosis factor alpha (TNF-α), interleukin 4 (IL-4), and interleukin 10 (IL-10).

## Data Availability

The data supporting the findings of this study are available from the corresponding author upon request.
